# Protein Concentrates on Tepary Bean (*Phaseolus acutifolius* Gray) as a Functional Ingredient: *In silico* Docking of Tepary Bean Lectin to Peroxisome Proliferator-Activated Receptor Gamma

**DOI:** 10.3389/fnut.2021.661463

**Published:** 2021-05-31

**Authors:** Cristina López-Ibarra, Felipe de Jesús Ruiz-López, Minerva Bautista-Villarreal, Juan Gabriel Báez-González, Beatriz Adriana Rodríguez Romero, Blanca Edelia González-Martínez, Manuel López-Cabanillas Lomelí, Jesús Alberto Vázquez-Rodríguez

**Affiliations:** ^1^Laboratorio de Alimentos, Centro de Investigación en Nutrición y Salud Pública, Facultad de Salud Pública y Nutrición, Universidad Autónoma de Nuevo León, Monterrey, Mexico; ^2^Departamento de Alimentos, Facultad de Ciencias Biológicas, Universidad Autónoma de Nuevo León, Monterrey, Mexico; ^3^Centro de Investigación y Desarrollo en la Industria Alimentaria, Facultad de Agronomía, Universidad Autónoma de Nuevo León, Monterrey, Mexico

**Keywords:** PPAR-gamma, underutilized food, tepary bean, *Phaseolus acutifolius*, *in silico* docking, vegetal protein

## Abstract

The tepary bean (*Phaseolus acutifolius* Gray) is a US–Mexico frontier native crop, produces high yields in agriculture, and needs to be reconsidered because of its nutritional and functional properties. This study aimed to determine the technological and nutritional properties of flours and protein concentrates of tepary bean, besides determining an *in silico* agonist effect of tepary bean lectin to peroxisome proliferator-activated receptor gamma (PPAR-γ). We evaluated the technological properties of raw samples (tepary flour and tepary protein concentrate) and cooked samples (tepary flour and tepary protein concentrate). The flours present a significant difference (*p* < 0.05) concerning protein concentrates in water absorption and oil absorption capacity. The raw samples' emulsifying capacity was higher than that reported in the literature for other legumes, but not the cooked samples. The samples' foaming capacity had no significant difference in treatments (*p* > 0.05), and cooked tepary bean protein concentrate presented complete gelation at a lower concentration (2%). Nutritionally, raw samples present a protein percentage of 23.46 ± 0.06 and 71.38 ± 0.44 and cooked samples present a protein percentage of 25.27 ± 0.04 and 62.69 ± 0.14; a chemical score of 72, 86, 82, and 72; *in vitro* protein digestibility (%) = 48.20 ± 0.31, 49.80 ± 0.80, 61.77 ± 1.70, and 63.61 ± 4.19; and C-PER = 0.86, 1.34, 1.93, and 1.81, respectively. All the samples showed methionine + cysteine as the limiting amino acid. All these nutritional data are very similar to the common bean (*Phaseolus vulgaris*). SDS-PAGE preserves the lectin fraction in both protein concentrates. The *in silico* study of tepary lectin (PDB: 6tt9) shows that there were seven peptides that presented values below −120 kcal/mol: PEW, VSVGF, PSQK, TTPW, ATSF, ITY, and TSF, with VSVGF, PSQK, and PEW having the highest affinity for active sites of the PAPRγ receptor (binding energies from −5.32 to −7.04 kcal/mol). These peptides could show antiadipogenic or antidiabetic activity based on the intermolecular bond energies and open an interesting research item.

## Introduction

The chemical composition of foods defines their structure, organoleptic properties, and bioavailability of macro- and micronutrients. Some foods such as grains and legumes can be processed into ingredients and used to develop new products. In recent years, the beneficial relationship of increased consumption of plant-based protein and the decrease or prevention of various pathologies such as obesity, cancer, and hypertension, compared to the intake of a typical omnivorous diet, has been ratified (1–4). Tepary bean (*Phaseolus acutifolius* Gray) is a species native to Mexico, grown mainly in the USA–México frontier and Sinaloa state, cultivated principally for self-consumption (5). *P. acutifolius* is an edible bean and naturally adapted to arid and semi-arid conditions. It is resistant to adverse agronomic conditions such as high concentrations of salt, limited water conditions, pests, and microorganisms that affect the common bean. Also, the tepary bean has similar nutritional and technological characteristics to cowpeas and chickpeas (6). Furthermore, tepary bean proteins, specifically lectin fraction, have shown functional effects vs. cancer lines *in vitro* and *in vivo* studies (7–9). Due to this, it is of utmost importance to study other possible beneficial health effects of tepary bean lectins, such as the effect on regulatory mechanisms of obesity, a global epidemic. One of the most studied mechanisms is that of peroxisome proliferation factors (PPARs), especially PPAR-γ. PPAR-γ is responsible for regulating the gene expression of enzymes involved in the storage of fatty acids in adipose tissue, such as acyl-CoA synthase, lipoprotein lipase, and phosphoenolpyruvate carboxykinase (10). PPAR-γ is principally involved in lipogenesis, adipocyte differentiation, cell proliferation, and insulin sensitivity (11). Recently, it has been seen as a therapeutic target for the treatment of metabolic disorders such as diabetes and dyslipidemia. However, the drugs have side effects such as edema and congestive heart failure (12); therefore, the search for natural agents that present an agonist effect in PPAR-γ is of utmost importance. This study aimed to determine the technological and nutritional properties of protein concentrates of tepary bean, besides determining an *in silico* agonist effect of tepary bean lectin to peroxisome proliferator-activated receptor gamma (PPAR-γ) for the identification of antiadipogenic compounds (13).

## Materials and Methods

### Biological Material

The seeds of white tepary bean (*P. acutifolius*) were cultivated and harvested in 2017 (Faculty of Agronomy of the Universidad Autónoma de Nuevo León). The dried beans were stored at 4°C and protected from a light until they are processed.

### Flour and Protein Concentrate Obtainment

For the production and analysis of cooked bean flour, 100 g of raw tepary bean seeds was cleaned and soaked in 200 mL of distilled water for 4 h at room temperature. Subsequently, the cooking process was at 100°C for 1 h. Once cooked, they were dried in the oven (Shel Lab model SMO3, Cornelius, USA) at 65°C for 4 h and ground (IKA model M20, Wilmington, USA). The dehydrated sample was ground and sieved through mesh number 20 (aperture: 850 μm) to obtain a homogeneous particle size; the sample (CF) was stored in vacuum in polyethylene bags at 4°C (6). The raw tepary bean seeds (RF) were milled (IKA model M20, Wilmington, USA) and went through the same mesh (14). The protein concentrates, cooked (CPC) and raw (RPC), were obtained through 14 with some modifications. We suspended flours (RF and CF) in distilled water at a concentration of 1:6 (w/v) and pH was adjusted to 11 (NaOH 1 M). Subsequently, the samples were agitated for 1 h and filtered through mesh number 80 (180 μm). The remaining suspension was adjusted to pH 4.5 with HCl (1 M) and centrifuged for 12 min at 2,200×*g* (Hermle model Z 326K, Wehingen, Germany); the precipitate was freeze-dried under conditions of −45°C for 48 h (Labconco model FreeZone 4.5 L, Kansas City, USA) and stored at 4°C.

### Physicochemical Characteristics

#### Water Absorption Capacity and Oil Absorption Capacity

Water absorption capacity (WAC) tests were reported according to Kaur and Singh (15). Three grams of each flour was dispersed in 25 mL of distilled water for 30 min with manual stirring, followed by a 25-min centrifugation period at 1,110×*g* (Hermle model Z 326K, Wehingen, Germany) at neutral pH and room temperature. We discard the supernatant, and the tubes were in the oven at 50°C for 25 min. The water absorption was measured in milliliters of water per gram of flour sample (mL/g sample). Finally, the sample was reweighed and analyzed. The oil absorption capacity (OAC) was according to Julianti et al. (16) with slight modifications. One gram of sample was suspended in 5 mL of corn oil in a centrifuge tube. The tube was shaken for 1 min at room temperature at neutral pH and then centrifuged at 1,110×*g* (Hermle model Z 326K, Wehingen, Germany) for 25 min. We discarded the supernatant and the samples were analyzed. The oil absorption was measured in milliliters of oil per gram of flour sample (mL/g sample).

#### Foaming Capacity

The foaming capacity is expressed as foam in milliliters per 100 mL of sample. Using a homogenizer (IKA model T-50, Wilmington, USA), 1 g of sample and 50 mL of distilled water were mixed into a 100-mL test piece for 5 min. The initial volume of the sample was measured at 0 min and 60 min, verifying the foam presence (17). The foaming capacity is expressed as foam in milliliters per 100 mL of test sample (mL/100 mL).

#### Emulsion Capacity

One gram of sample with 20 mL of distilled water was mixed at vortex agitation (Labnet model VX-200, Tewksbury, USA) for 15 s in a graduated tube of 50 mL. Later, the pH was adjusted to 7 with NaOH (0.1 N) and HCl (0.1 N), and a volume of 25 mL of distilled water and 25 mL of edible soybean vegetable oil were added and finally shaken for 3 min with a homogenizer (IKA model T-50, Wilmington, USA) and centrifuged at 210×*g* (Hermle model Z 326K, Wehingen, Germany) for 5 min. The emulsion was expressed in mL/100 mL as the emulsified layer's height concerning the total liquid (18).

#### Gelation Capacity

In glass tubes with a lid, the sample suspensions [sample concentration of 2, 4, 6, 8, 10, 12, 14, and 16% in distilled water (w/v)] were placed in a water bath at 100°C for 1 h and then allowed to cool to 4°C for 2 h. The results were taken when the tubes were inverted and recorded as complete gelling (+), no gelling (–), and partial gelling (±) (19).

### Chemical and Nutritional Characteristics

#### Proximate Composition

Moisture (925.10), protein (920.06), ash (936.07), dietary fiber (985.29), and fat (920.09) were determined following the approved method of the American Association of Cereal Chemists (20). Carbohydrate was calculated by the difference and energy was calculated by multiplying the protein and carbohydrate by 4 and fat by 9, respectively. The determination of the amino acid score of the samples (CF, RF, CPC, and RPC) was carried out through the method of Vázquez-Ortiz et al. (21). Tryptophan was identified using the method of Dávila and Martínez with Betancur-Ancona modifications (14).

#### Chemical Score

The most limiting essential amino acid (EAA) in the sample was identified, for which the content of each EAA was compared with that recommended by FAO (3 years and older) (20, 22). The CS was calculated as follows:

$$\begin{array}{l} CS=\frac{\text{Content of the most limiting EAA}}{\text{REAAR}}\;\times \;100 \end{array}$$

Where, CS = chemical score, EAA = essential amino acid, and REAAR = recommended EAA requirement.

#### Calculated Protein Efficiency Ratio

The C-PER was evaluated according to the AOAC methodology (20). This procedure was based on the *in vitro* protein digestibility (GIS) and the EAA composition of the different samples (23).

#### *In vitro* Digestibility

Simulation of human gastrointestinal digestion (GIS) was conducted *in vitro* as reported Shim et al. and Xing et al. with some modifications (24, 25). The whole GIS digestion steps were performed sequentially at three phases, at 37°C. The pH was adjusted with HCl or NaOH (4 M) during digestion. We take aliquots at

1. before digestion (0 min);
2. after 1, 5, 30, and 60 min of gastric phase; and
3. after 1, 5, 30, and 120 min of duodenal phase.

After each digestion time, the digested samples were filled with deionized water to 14 mL and then heated in boiling water for 5 min to stop the enzymatic digestions. The digested samples were centrifuged (10,000×*g*, 20 min, 4°C) except those prepared for particle size distribution. The supernatants were kept frozen at −20°C until use to determine the protein digestibility (%) for Dumas methodology (920.06) (20) and calculated by:

$$\begin{array}{l} IVPD=\frac{\%FP\;}{\%IP}\times 100 \end{array}$$

Where IVPD is *in vitro* protein digestibility, FP is protein percentage at 120 min duodenal phase, and IP is protein percentage at 0 min.

##### Particle Size Distribution

The analysis of the particle size distribution of the samples from each digestion period was measured by an integrated laser light scattering instrument (Malvern Panalytical, model Mastersizer 3000, Southborough, USA). A value of 1.33 for water was used as a refractive index for the continuous phase. The particle size values were measured as D [4,3] and D [v,0.90], reported to describe 90% of the particles' total number (25).

#### Electrophoresis (SDS-PAGE)

The total protein of CPC and RPC concentrates was analyzed by denaturing electrophoresis in polyacrylamide gels (SDS-PAGE). Following the method proposed by Laemmli with some modifications (26): 10 mg of each sample was dissolved in 1 mL of sodium dodecyl sulfate solution (SDS, 1% w/v). Five microliters of BenchMark Protein Ladder^TM^ was the molecular weight marker. The proteins were separated (Bio-Rad Mini-PROTEAN model 1658000EDM, Hercules, USA) by a vertical electrophoresis chamber in Mini Geles Teo-Tricine SDS 4–12% RunBlue^TM^ for 2 h, at 80 V (1 h) and 100 V (1 h). The separated proteins were stained with staining solution Coomassie R-250 blue (0.1%), 40% methanol, and 10% glacial acetic acid, and gentle stirring for 2.5 h, then faded with a methanol solution (40%). The images were photo-documented and analyzed.

### *In silico* Methodology

#### *In silico* Digestibility

The sequence of the lectin protein fraction of tepary bean (*P. acutifolius*) was from the Protein Data Bank of Europe (https://www.ebi.ac.uk/pdbe/entry/pdb/6tt9/protein/1) (Table 1). *In silico* hydrolysis process was according to Shi et al. (27). It was performed through the BIOPEP UWM protein database using the digestive proteolytic enzymes pepsin (EC 3.4.23.1), trypsin (EC 3.4.21.4), and chymotrypsin (EC 3.4.21.1).

**Table 1:** The amino acid sequence of the lectin protein in FASTA format from tepary bean (*Phaseolus acutifolius*).

EAEAAASANDISFNFQRFNETNLILQGDASVSSSGQLRLTNLNDNGEPTLSSLGRAFYSTPIQI
WDSTTGAVASFATSFTFNIRVPNNAGPADGLAFALVPVGSKPKDRGGLLGLFDGSDSKAHT
VAVEFDTLYNRDWDPRERHIGIDVNSIKSIKTTPWDFVNGEDAEVLITYDSSTKLLVASLVYP
SQKTSFIVSDTVDLKSVLPEWVSVGFSATSGISKGNVETNDLLSWSFASKLSDGTTSEGLNHH
HHHH

#### Ligand Preparation

Peptide preparation was according to Ye et al. (28) with some modifications. The peptides released from the digestive process with lengths between two and five amino acid residues were used for the subsequent tests. First, HPEPDOCK software evaluated the ligand-receptor affinity, considering binding energy of <−120 kcal/mol as high affinity. Later, these high-affinity peptide ligands were converted to SMILES (Simplified molecular-input line-entry system) format using the Dendrimer Builder tool (https://dendrimerbuilder.gdb.tools/) and the Avogadro software to model them, and finally, there was subsequent identification of *in silico* antiadipogenic activity at the PPARγ receptor. As a control, we used the G3335 and GW9662 molecules. The sequences were from the PubChem database (https://pubchem.ncbi.nlm.nih.gov/).

#### Receptor Preparation

**Figure 1:**
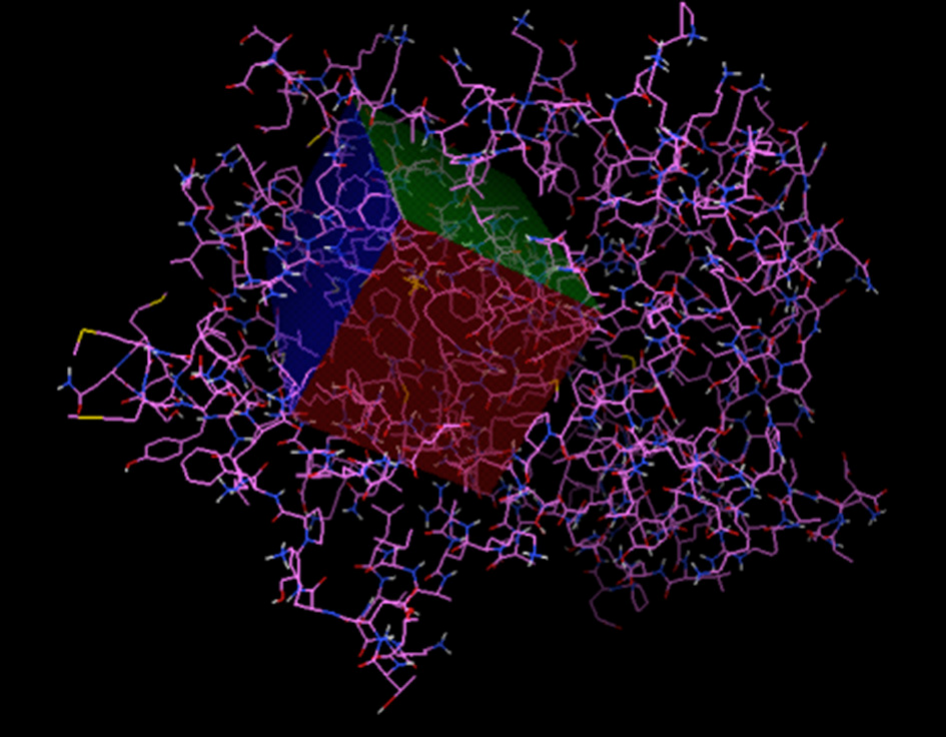
The active region of PPARγ.

The PPARγ receptor was extracted in crystallized form from the PDB protein database with the code 3V9V (https://www.rcsb.org/structure/3V9V) (Figure 1). This protein binds to specific regions of the DNA involved, mainly in the process of adipocyte differentiation, in the form of a heterodimer with the retinoid X receptor (RXR). We use the Autodock tools program for the affinity analysis of the ligands in the receptor, with an interaction area centered at 7,745 × 50,606 × 57,552 and with dimensions of *X* = 70, *Y* = 40, and *Z* = 40 with a spacing of 0.375 angstroms (Figure 1) to cover those regions of the PPARγ receptor involved with antiadipogenic effects (Phe264, His266, Ile281, Cys285, Arg288, Ser289, Met348, and His449) (28, 29).

### Statistical Analysis

Data were expressed as mean ± SD. One-way analysis of variance (ANOVA) was used, and comparison of means was done using Tukey's *post hoc* test (Minitab version 17, Minitab Inc., State College, PA, USA), and two-factor ANOVA was applied to particle size distribution assay. Means were considered significantly different at *p* ≤ 0.05. All the analyses were made in triplicate except chemical score, C-PER, *in silico* analysis (calculated), and particle size distribution (*n* = 10).

## Results

### Physicochemical Characteristics

#### WAC and OAC

The WAC of tepary bean flours at room temperature and neutral pH in RF and CF samples (2.40 ± 0.20 and 2.40 ± 0.20 mL/g sample) and RPC and CPC (1.40 ± 0.00 and 1.46 ± 0.11 mL/g sample) presented a significant difference (*p* < 0.05) between flours and protein concentrates (Table 2). The opposite occurred with OAC in flour samples (1.26 ± 0.40 and 1.20 ± 0.00 mL/g sample) vs. protein concentrate samples (1.46 ± 0.20 and 1.80 ± 0.40 mL/g sample), showing a significant difference (*p* ≤ 0.05) with respect to flour samples.

**Table 2:** Physicochemical properties of tepary bean flours (RF and CF) and protein concentrates (RPC and CPC).

**Samples**	**WAC (mL/g samples)**	**OAC (mL/g samples)**	**Foaming capacity (mL/100 mL)**	**Emulsion capacity (mL/100 mL)**
RF	2.40 ± 0.20^b^	1.26 ± 0.40^a^	0.50 ± 0.50^a^	100.03 ± 0.10^c^
CF	2.40 ± 0.20^b^	1.20 ± 0.20^a^	0.50 ± 0.50^a^	49.33 ± 6.42^a^
RPC	1.40 ± 0.00^a^	1.46 ± 0.20^b^	0.50 ± 0.50^a^	94.66 ± 9.23^c^
CPC	1.46 ± 0.11^a^	1.80 ± 0.40^c^	0.50 ± 0.50^a^	68.03 ± 4.05^b^

*RF, raw flour; CF, cooked flour; RPC, raw protein concentrate; CPC, cooked protein concentrate*.

*WAC, water absorption capacity; OAC, oil absorption capacity*.

*Data presented as the mean ± SD. Means with different superscripts in columns differ significantly (p ≤ 0.05), n = 3*.

#### Foaming Capacity

All the samples showed low foaming capacity and stability, with 0.50 mL/100 mL ± 0.50, with no significant difference (*p* > 0.05) (Table 2).

#### Emulsion Capacity

RF and RPC (100.03 ± 0.10 and 94.66 ± 9.23 mL/100 mL) had higher emulsifying capacity than CF and CPC samples (49.33 ± 6.42 and 68.03 ± 4.05 mL/100 mL) (Table 2), presenting a significant difference (*p* ≤ 0.05) between raw and cooked sample groups (Table 2).

#### Gelation Capacity

CF needed a lower percentage for gelation (2%) and preserved this capacity from 2 to 16%. RF and CPC presented gelation from 8 to 16% and from 12 to 16%, respectively. RF, CPC, and RPC presented partial gelation from 2 to 6%, 2 to 10%, and 6 to 16%, respectively (Table 3).

**Table 3:** Results of gel formation of tepary bean flours (RF and CF) and protein concentrates (RPC and CPC).

**Samples**	**Concentration (% w/v)**							
	**2**	**4**	**6**	**8**	**10**	**12**	**14**	**16**
RF	±	±	±	+	+	+	+	+
CF	+	+	+	+	+	+	+	+
RPC	–	–	±	±	±	±	±	±
CPC	±	±	±	±	±	+	+	+

*RF, raw flour; CF, cooked flour; RPC, raw protein concentrate; CPC, cooked protein concentrate*.

*(±) = partial gel formation, (+) = complete gel formation, (–) = no gel formation, n = 3*.

### Chemical and Nutritional Characteristics

#### Proximate Composition and Nutritional Properties

RF and CF presented the lowest values for energy, total protein, and total lipids and the highest values for total carbohydrates, with no significant difference (*p* > 0.05) between them. RPC showed the highest value for total protein (71.38 ± 0.44%), with a significant difference (*p* < 0.05) from the other samples. CPC presented the highest value of energy (413.85 + 0.91 kcal). All the samples were significantly different (*p* < 0.05) in energy (Table 4). The limiting EAAA in the four samples was sulfur amino acids (methionine, cysteine), and the C-PER was higher in cooked samples (CF and CPC), with 1.93 and 1.81 *vs*. 0.86 and 1.34 in raw samples (RF and RPC), respectively (Table 5).

**Table 4:** Proximal chemical composition of tepary bean flours (RF and CF) and protein concentrates (RPC and CPC)^*^.

**Samples/Proximal chemical composition**	**Total protein (%)**	**Total lipids (%)**	**Dietary fiber (%)**	**Total carbohydrates (%)**	**Energy (kcal)**
RF	23.46 ± 0.06^a^	1.89 ± 0.79^a^	2.96 ± 0.70^a^	66.02 ± 0.04^c^	374.93 ± 0.88^a^
CF	25.27 ± 0.04^a^	1.09 ± 0.02^a^	2.44 ± 0.36^a^	68.35 ± 1.10^c^	384.29 ± 1.16^b^
RPC	71.38 ± 0.44^c^	5.12 ± 0.03^b^	ND^+^	15.59 ± 0.52^a^	393.96 ± 1.32^c^
CPC	62.69± 0.14^b^	9.97 ± 0.29^c^	ND^+^	18.34 ± 0.22^b^	413.85 + 0.91^d^

*RF, raw flour; CF, cooked flour; RPC, raw protein concentrate; CPC, cooked protein concentrate*.

* *Reported on dry basis*.

*Data presented as the mean ± SD*.

+ *Not determined*.

*Means with different superscripts in columns differ significantly (p < 0.05), n = 3*.

**Table 5:** Amino acid composition and nutritional evaluation of tepary bean flours (RF and CF) and protein concentrates (RPC and CPC).

**Amino acid (g/100 g protein)**	**RF**	**CF**	**RPC**	**CPC**	**FAO Ref*^+^***
Asp	7.64 ± 0.03*^d^*	7.50 ± 0.02*^c^*	6.83 ± 0.01*^b^*	6.50 ± 0.03*^a^*	–
Glu	13.54 ± 0.01*^b^*	11.05 ± 0.05*^a^*	13.71 ± 0.01*^c^*	15.13 ± 0.02*^d^*	–
Ser	1.42 ± 0.04*^b^*	1.86 ± 0.02*^c^*	1.46 ± 0.04*^b^*	0.90 ± 0.01*^a^*	–
His	5.51 ± 0.03*^b^*	5.02 ± 0.01*^a^*	6.23 ± 0.01*^c^*	8.49 ± 0.05*^d^*	1.6
Gly	3.67 ± 0.04*^b^*	3.16 ± 0.03*^a^*	4.31 ± 0.02*^d^*	4.23 ± 0.01*^c^*	–
Thr	7.56 ± 0.01*^a^*	8.46 ± 0.04*^b^*	12.13 ± 0.03*^d^*	10.61 ± 0.04*^c^*	2.5
Arg	5.45 ± 0.02*^d^*	4.04 ± 0.03*^b^*	4.96 ± 0.04*^c^*	3.69 ± 0.02*^a^*	–
Ala	2.50 ± 0.03*^a^*	2.80 ± 0.04*^b^*	3.95 ± 0.02*^d^*	3.10 ± 0.04*^c^*	–
Meth+Cys	1.65 ± 0.02*^a^*	1.99 ± 0.04*^c^*	1.89 ± 0.03*^b^*	1.65 ± 0.02*^a^*	2.3
Val	8.90 ± 0.02*^d^*	6.13 ± 0.01*^a^*	6.31 ± 0.05*^b^*	7.30 ± 0.04*^c^*	4.0
Phe+Tyr	11.99 ± 0.03*^c^*	10.63 ± 0.03*^b^*	8.77 ± 0.04*^a^*	11.97 ± 0.03*^c^*	4.1
Ile	4.18 ± 0.03*^b^*	3.97 ± 0.02*^a^*	4.59 ± 0.02*^c^*	4.75 ± 0.03*^d^*	3.0
Leu	6.25 ± 0.03*^a^*	6.47 ± 0.01*^b^*	7.31 ± 0.03*^c^*	7.92 ± 0.02*^d^*	6.1
Thr	7.56 ± 0.01*^a^*	8.46 ± 0.02*^b^*	12.13 ± 0.03*^d^*	10.61 ± 0.03*^c^*	2.5
Trp	1.10 ± 0.05*^b^*	1.20 ± 0.03*^c^*	0.90 ± 0.02*^a^*	1.20 ± 0.01*^c^*	0.66
Lys	3.48 ± 0.04*^b^*	4.34 ± 0.02*^c^*	1.89 ± 0.04*^a^*	4.86 ± 0.03*^d^*	4.8
EAA chemical score	72	87	82	72	100
Limiting EAA	Meth+Cys	Meth+Cys	Meth+Cys	Meth+Cys	
Total protein (%)	*23.46 ± 0.06^a^*	*25.27 ± 0.04^b^*	*71.38 ± 0.44^d^*	*62.69± 0.14^c^*	
IVPD (%)	48.20 ± 0.71*^a^*	61.77 ± 1.70*^b^*	49.80 ± 0.80*^a^*	63.61 ± 4.19*^b^*	
C-PER	0.86	1.93	1.34	1.81	

*RF, raw flour; CF, cooked flour; RPC, raw protein concentrate; CPC, cooked protein concentrate*.

*EAA, Essential amino acid (g/kg protein). IVPD, in vitro protein digestibility. C-PER, calculated protein efficient ratio. Means with different superscripts in the same row are different (p ≤ 0.05), n = 3*.

+ * = (30)*.

#### GIS and Particle Size Distribution

% IVPD of CF and CPC (61.77 ± 1.70 and 63.61 ± 4.19) are higher than those of RF and RPC (48.20 ± 0.31 and 49.90 ± 0.80), showing a significant difference (*p* < 0.05); however, the CPC and CF's proximal analysis showed a significant difference (*p* < 0.05) in the protein percentage (Table 5). The % IVPD difference between RPC and CPC had a variation of 13.81%. The values of 90% of the particles at the duodenal phase for RF and RPC (442.60 ± 1.14 μm and 46.86 ± 0.83 μm) concerning CF and CPC (150.40 ± 0.89 μm and 42.46 ± 0.32 μm) showed that although the flours had a significant difference between treatments (*p* < 0.05), the protein concentrates did not show a significant difference (*p* > 0.05) (Table 6).

Table 6Particle size distribution (μm) of 90% of total particles while *in vitro* protein digestion (IVPD).

**Table:** 

	**Gastric phase**				
**Sample/Time (min)**	**0**	**1**	**5**	**30**	**60**
RF	390.90 ± 4.65^d^^C^	355.80 ± 1.92^c^^B^	328.30 ± 12.84^c^^A^	481.80 ± 1.30^d^^E^	452.20 ± 0.83^c^^D^
CF	269.80 ± 5.18^c^^C^	296.80 ± 0.83^d^^D^	286.20 ± 9.80^d^^D^	221.00 ± 1.58^c^^A^	227.20 ± 1.64^b^^B^
RPC	243.30 ± 2.83^b^^E^	7.40 ± 0.16^a^^A^	21.78 ± 2.16^a^^B^	92.02 ± 1.41^a^^C^	111.00 ± 5.49^a^^D^
CPC	140.90 ± 8.90^a^^C^	129.40 ± 2.07^b^^B^	122.30 ± 4.78^b^^B^	118.60 ± 1.34^b^^B^	105.60 ± 0.54^a^^A^

**Table:** 

	**Duodenal phase**			
**Sample/Time (min)**	**1**	**5**	**30**	**120**
RF	364.00 ± 1.00^c^^A^	478.40 ± 1.51^d^^B^	473.20 ± 2.68^d^^B^	442.60 ± 1.14^c^^B^
CF	172.00 ± 1.22^b^^C^	176.60 ± 0.54^c^^C^	162.40 ± 1.81^c^^B^	150.40 ± 0.89^b^^A^
RPC	21.04 ± 0.62^a^^A^	54.62 ± 0.83^a^^C^	59.46 ± 1.15^a^^C^	46.86 ± 0.83^a^^B^
CPC	77.12 ± 0.54^a^^D^	68.10 ± 0.62^b^^C^	50.44 ± 0.36^b^^B^	42.46 ± 0.32^a^^A^

*RF, raw flour; CF, cooked flour; RPC, raw protein concentrate; CPC, cooked protein concentrate*.

*Data are expressed as means ± SD, n = 10*.

a–d *Means with different superscripts in columns differ significantly (p < 0.05)*.

A−E *Means with different superscripts in rows differ significantly (p < 0.05)*.

#### Electrophoresis (SDS-PAGE)

**Figure 2:**
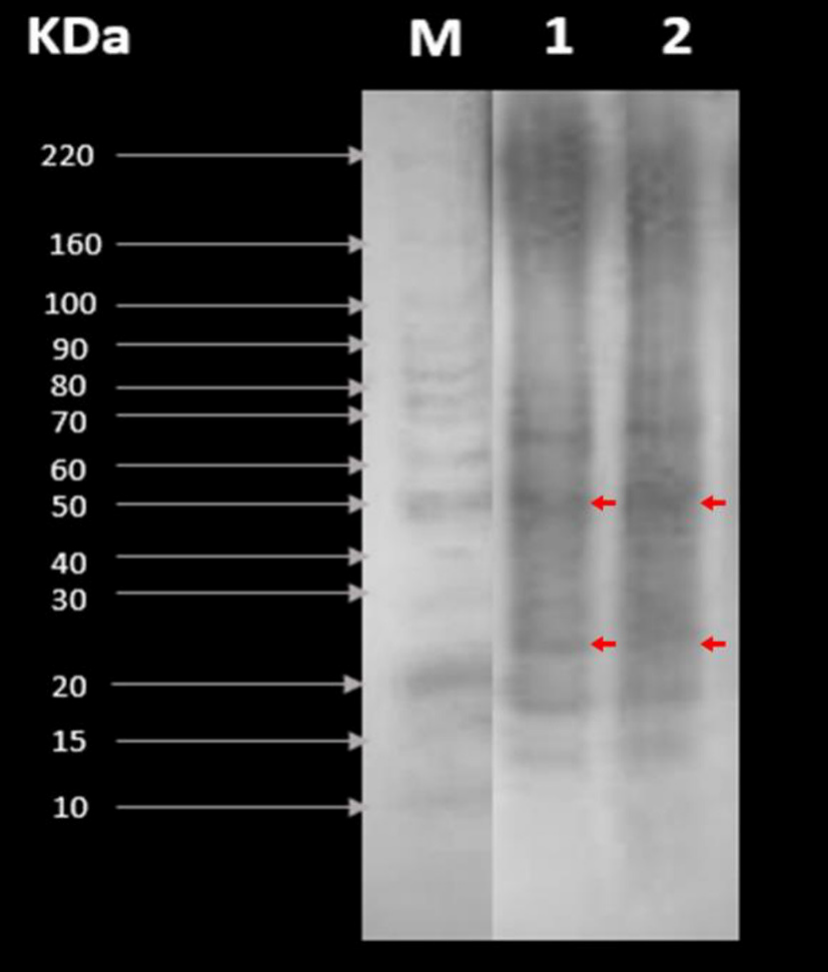
SDS-PAGE patterns of (1) raw protein concentrate (RPC) and (2) cooked protein concentrate CPC. M is the molecular weight marker (control).

Figure 2 shows the tepary bean proteins (RPC and CPC) separated by molecular weight in the presence of SDS. We could observe that the predominant proteins are those whose molecular weight is 50 kDa (phaseolin fraction) and between 20 and 30 kDa (lectin subunit fraction). Also, it showed smaller bands at 15–20 (albumin + globulin), 30–50 (gliadin), and 70–80 (high-molecular-weight glutenin subunit) kDa. Both samples showed electrophoretic patterns and behavior similar to concentrations of 5 μg/μL protein.

### *In silico* Methodology

#### *In silico* Digestibility

**Figure 3:**
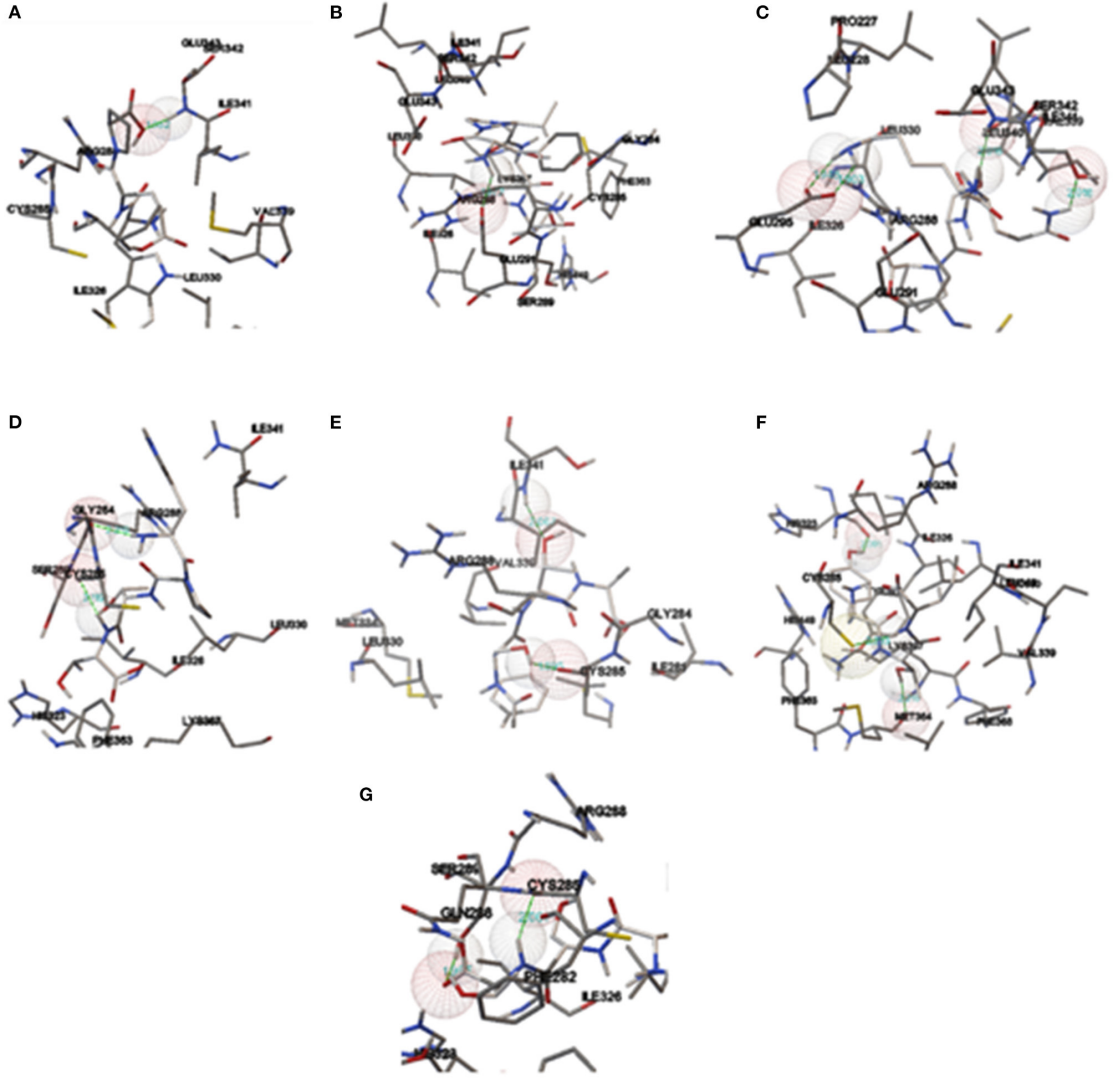
Intermolecular ligand-receptor conjunctions between PPARγ and peptides. **(A)** ITY, **(B)** TSF, **(C)** PEW, **(D)** PSQK, **(E)** TTPW, **(F)** ATSF, and **(G)** VSVGF.

The proteolytic process used the BIOPEP UWM protein database using the proteolytic enzymes pepsin, trypsin, and chymotrypsin. The peptides released from the proteolytic process are shown in Table 7, in which the peptides with a length between two and five amino acids were the most significant amount released (51.13%), followed by the free amino acids (34.09%), and finally, the peptides with a length of >5 amino acids had the least amount (14.77%). Subsequently, the peptides released from the digestive process with a length between two and five amino acids were evaluated for their affinity score toward the PPARγ receptor using the HPEPEDOCK SERVER® program. There were 45 peptides analyzed, obtaining values between −50.73 and −157.90 kcal/mol, with 7 peptides that had values below −120 kcal/mol, which are high-affinity peptides toward PPARγ (Table 8). Finally, the binding analysis of high-affinity peptides with PPARγ anti-adipogenic regions was carried out with the Autodock tools® program (Figure 3). The results obtained show that the tripeptide PEW, ITY, and TSF obtained a binding energy of −6.38, −5.91, and −4.68 kcal/mol, respectively; the binding sites for PEW were at Ser342; those for ITY were at Met364, His323, and Cys285; and those for the TSF tripeptide were at Cys285 and Ser289. The tetrapeptide PSQK, TTPW, and ATSF, with an energy of −7.04, −5.96, and −3.89 kcal/mol, respectively, were obtained with bonds Ser342, Ile262, and Glu291 for PSQK; Cys285 and Gly284 for TTPW; and Cys285 and Ser342 for the TSF peptide. Finally, the pentapeptide VSVGF obtained a binding energy of −5.97 kcal/mol toward the Glu291 residue (Table 9).

**Table 7:** *In silico* hydrolysis of the lectin protein.

**Length of peptides**	**Number of peptides**	**%**
1	30	34.09
2–5	45	51.13
>5	13	14.77

**Table 8:** Ligand-receptor affinity scores obtained with HPEPDOCK SERVER (kcal/mol).

**Protein**	**Results of the linkage evaluation by HPEPDOCK (kcal/mol)**
Lectin	PEW [−157.801], VSVGF [−157.768], PSQK [−152.549], TTPW [−145.896], ATSF
	[−137.604], ITY [−126.941], TSF [−123.304], DISF [−113.395], VY [−112.665], SW
	[−111.589], VASL [−110.73], T F [−103.79], DSSTK [−101.083], VPN [−100.335],
	IL [−95.602], VETN [−94.906], GEPTL [−94.454], DW [−93.887], SF [−93.527],
	SVL [−92.453], QR [−92.11], PK [−91.866], AF [−91.54], AF [−91.54], SIK
	[−89.499], SIK [−89.499], IR [−89.461], DPR [−89], SSL [−87.756], ETN
	[−87.603], AH [−85.115], DR [−82.929], GR [−82.866], ER [−81.692], DF
	[−78.98], ASK [−78.633], TN [−77.845], DTL [−77.331], GGL [−72.523], VN
	[−71.853], GN [−63.773], DN [−62.512], AL [−61.361], GL [−53.133], DL
	[−50.735]

**Table 9:** Binding energies and bonds of peptides with binding point <−120 kcal/mol in antiadipogenic sites of PPARγ.

**Peptide**	**Length**	**Score binding kcal/mol (HPEPDOCK)**	**Binding energy**	**Interaction**
ITY	3	−126.941	−5.91	Met364 His323 Cys285
TSF		−123.304	−4.68	Cys285 Ser289
PEW		−157.801	−6.38	Ser342
PSQK	4	−152.549	−7.04	Ser342 Ile 262 Glu291
TTPW		−145.896	−5.96	Cys285 Gly284
ATSF		−137.604	−3.89	Cys285 Ser342
VSVGF	5	−157.768	−5.97	Glu291

## Discussion

### Physicochemical Characteristics

#### WAC and OAC

The WAC of tepary bean flours at room temperature and neutral pH presented values similar to the varieties Negro 8025 and Bayo Madero of common bean (*Phaseolus vulgaris*) reported by Ramírez et al. (31), which had a range of 2.17 to 2.58 mL/g sample. Those samples were cooked and subsequently dehydrated. RPC and CPC had higher values than those reported by Butt and Rizwana (32) for protein isolate of guandu bean (*Cajanus cajan*) and caupi (*Vigna unguiculata*) (0.97 ± 0.04 and 1.38 ± 0.09 mL/g sample), but less than those of mung bean (*Vigna radiata*) and pea (*Pisum sativum*) (1.63 ± 0.10 and 1.52 ± 0.09 mL/g sample, respectively). The WAC of legumes is a property related to heat treatment, which reduces or increases the speed and amount of hydration, positively affecting the viscosity but could promote microbial growth (31, 33). Besides, the presence of hydrophilic carbohydrates could be responsible for increasing this parameter in flours, compared to the reported values for protein isolates and concentrates. In those, the number of components (protein, starch, fiber) allows to absorb and retain more significant amounts of water (34) and the conformal characteristics and availability of polar amino acids (32). OAC for RF and CF values was lower than that of cargamanto bean and black bean (1.48 ± 0.09 and 1.34 ± 0.11 mL/g sample) (35), but greater than that reported by Ramirez et al. (31) for the Varieties Black 8025 and Bayo Madero of common bean (0.76 to 0.88 mL/g). Butt and Rizwana (32) reported values similar to those obtained for RPC and CPC samples in pea isolates (*P. sativum*) (1.40 ± 0.08 mL/g sample) and caupi bean (*V. unguiculata*) (1.45 ± 0.06 mL/g sample). OAC is an essential physicochemical property in food processing and food storage; it influences the entrance and development of oxidative power, determining consumer acceptance and product quality and could be a determinant for hydrophilic groups and polar amino acids on the surface of protein molecules (32, 33).

#### Foaming Capacity

The results (Table 2) were higher for RF and RPC and similar for CF and CPC samples reported by Siddiq et al. (35) for red kidney, small red kidney, cranberry, and black bean flours (45.7, 38.2, 49.6, and 37.4 mL/100 mL, respectively). They mention factors that affect foaming capacity, such as flour composition, flour particle size, conditions of the process, and interactions between proteins and carbohydrates and between proteins and lipids. The tepary bean's low foaming capacity may be due to the type of protein, a characteristic that could represent an advantage that could increase the quantity and quality of protein in foods without changing their consistency (32, 36).

#### Emulsion Capacity

RF and RPC had higher emulsifying capacity than CF and CPC samples (Table 2), which resemble the values reported by Siddiq et al. (35) for black bean flour (45.6 ± 1.8 mL/100 mL). In other research, caupi bean protein isolates and chickpea showed lower emulsifying capacities than tepary bean after cooking (flour and protein concentrate) (33). The emulsifying ability is related to the potential to absorb water and oil, reflecting proteins' ability to impart stress resistance and changes over a defined period (33). Heat treatment influences the reduction of this capacity, justifying that raw samples have higher results (19, 34). Also, the increased emulsifying activity of tepary bean samples could be due to the difference in the chemical composition and solubility of the protein, as well as the dissociation and partial deployment of globular proteins, which exposes residues of hydrophobic amino acids and consequently increases surface activity and adsorption in the water and oil interface (33).

#### Gelation Capacity

Results were lower than other results reported in samples of cargamanto bean flour and black bean, pea protein, or caupi bean isolates (32, 35). Those results differ from research conducted by Gupta et al. (37), where tepary bean varieties did not present gel formation. Gelation capacity is a qualitative parameter that expresses the minimum concentration of protein at which the gel does not slide through the walls of the test tube in an inverted position, affecting the gel and thickness characteristics (38) due to permeability of seed by the thermal process (39). The lower the concentration required, the better the gelling capacity of proteins (35). Gelation capacity is an essential property in puddings, jellies, and batters for fried products (37).

### Chemical and Nutritional Characteristics

#### Proximate Composition and Nutritional Properties

RF and CF showed values similar to those reported by Idouraine et al. (40) for different varieties of tepary beans that grew in the southwestern United States of America and northern Mexico (Table 4). The percentage of protein (23.46 ± 0.06 to 25.27 ± 0.04%) and the rest of the parameters evaluated are in the general range for legumes and other beans varieties, as reported by the Spanish Nutrition Foundation (41). The protein, carbohydrates, and lipids of average cooked bean are below those of the tepary bean, which directly affects the energy content. On the other hand, the percentage of protein in both concentrates was similar to that reported by Betancur-Ancona et al. (14) for *Phaseolus lunatus*. The presence of sulfured amino acids as a limiting amino acid and the C-PER between 0.86 and 1.93 are similar to those reported in a wide range of legumes and pulses (42). These classify the cooked samples (CF and CPC) as medium-quality protein. The fact that the C-PER relates the GIS value and the EAA means that it may also indicate that the thermal and chemical processing made the samples more accessible to enzymatic action by denaturing (23).

#### GIS and Particle Size Distribution

The denaturation of the protein and the reduction in amino acid availability are crucial in their evaluation. Proteins could be affected by cross-linking, racemization, and degradation, and formation of complexes with sugar may result in loss of digestibility (43). IVPD and C-PER were similar to those reported by tepary bean in Idouraine et al. and Salas-López et al. (44–46) but not in limiting EAAA. Also, they showed similar values to common bean varieties like black, navy, pinto, and red kidney and fava bean with extrusion, baked, or traditional cooking process (47). These values would increase with a mix of other pulses or grains as QPM maize (43). Samples of raw tepary beans (RF and RPC) showed higher degrees of complexity during digestibility and particle sizes more extensive than those of thermally treated samples (CF and CPC). It was believed that the variation between particle size distribution could explain the enzyme–protein and phenolic compound–protein interaction, even more when tepary bean presents around 110 mg of total polyphenols (GAE/100 g dry sample) (48) and its phenolic compound profile is not completely known (49). Hydrogen bonding and hydrophobic interactions are responsible for the complex formation of protein and phenolics (50), and the pH variations and enzymatic activity could interact with the particle size through time (25). Therefore, depending on the source and processing, proteins can exhibit a wide range of heterogeneous and complex structures in the foods we consume. Also, because proteins have regions with different affinity for hydrophobic and hydrophilic environments, native molecular structures affect susceptibility to proteolysis (39).

#### Electrophoresis (SDS-PAGE)

The phaseolin fraction usually represents the largest proportion of proteins in grains and legumes (36–46%), with those inside the beans being the most abundant. On the other hand, lectins are a 120-kDa tetramer (subunits around 31 kDa) and represent 5–12% of the total protein in the genus *Phaseolus*. These have numerous functions in the plant (nitrogen fixation and protection against pathogens, among others) and, within their functional properties in animals, have been reported to have positive effects in the circulatory system, mainly against aging and tumor cells (7–9). This effect of lectins is due to the fact that they can recognize carbohydrates with high specificity of the cell surface or in suspension, agglutinating cells, and precipitate glycoconjugates. Regardless of their specificity, legume lectins recognize carbohydrates thanks to the presence of three amino acids: aspartic acid, asparagine, and an aromatic residue or leucine creating strong interactions between proteins and carbohydrates that could have an impact on WAC and OAC and GIS (51). It is also important to note that tepary bean lectin and its subunits are the only protein of *P. acutifolius* deposited within the Protein Data Bank (PDB: 6TT9), which opens up research opportunities on our native plant species.

### *In silico* Methodology

#### *In silico* Digestibility

Protein resistance to degradation and transport through the intestinal wall are two factors that influence the bioavailability of proteins, in which the degree of absorption is a function of the length of the peptide amino acid chain. Although information indicates that complete proteins' absorption could be through intercellular junctions, the highest absorption is from peptides with a length between two and five amino acid residues. The absorption of dipeptides and tripeptides is performed using PepT1 and PepT2 (SLC15A1, proton-dependent oligopeptide transporter), and for peptides with a length between four and five amino acids, absorption is carried out by SOPT1/SOPT2. Also, peptides with this length have a higher resistance to degradation, increasing their bioavailability and, more significantly, bioactivity (52). It has been reported that the determination of binding energy based on *in silico* experimental methods using HPEPDOCK shows a good correlation with the results obtained experimentally so that these molecules are the ones with the higher antiadipogenic potentiality (53, 54). However, concerning the results obtained with Autodock tools, the peptides TTPW, ATSF, ITY, and TSF showed the formation of hydrogen bonds in anti-adipogenic regions of the PPARγ receptor, mainly the three presented bonds with Cys285, which has been since it presents more significant antiadipogenic effects, with binding energies of −5.96, 3.89, −5.91, and −4.68 kcal/mol, respectively (28). The molecules GW9662 and G3335, which are widely known for their anti-adipogenic effect via PPARγ (55, 56), showed the formation of hydrogen bonds to Ser289 and Cys285 for GW9662 and Tyr473, Met364, His323, and Ser289 for G3335, with binding energies of −7.98 and −5.64 kcal/mol, respectively, for which the TTPW and ITY peptides are the ones that could present this antiadipogenic effect taking as reference the energies and binding sites of the controls used. Also, it is essential to consider the presence of hydrophobic amino acids (V, L, I, A, F, W, M, and P) since it has been reported that hydrophobic interactions is crucial for their functionality as bioactive compounds (57, 58). Oseguera Toledo et al. (59) have reported that the peptides FFL, LLSL, and LVLL, with a high content of hydrophobic amino acids, present a 13–28% inhibition in lipid accumulation. Therefore, the molecules must present this type of amino acids since their antagonistic mechanism via PPARγ is in adipose tissue (60). Also, it has been reported that the presence of proline in peptides shows higher resistance to degradation with intracellular peptidases, so that the tetrapeptide TTPW has a greater probability of being absorbed intact (61), and recently, Vega-Rojas et al. (62) demonstrated that derivate peptides from recombinant tepary bean lectin can cross through the intestinal membrane into *ex vivo* study with enterocytes. Nevertheless, different compounds found in the food matrix could be linked to peptide compounds, affecting their bioavailability, bioaccessibility, or bioactivity. One of the most studied is phenolic compounds; they could exert antagonistic or synergistic effects with proteins' bioactivity in their pure state (63, 64) and the presence of them in tepary bean protein concentrates would modify this results. Although PPARγ is the main protein for identifying antiadipogenic ligands, it is crucial to consider more proteins involved in this process, such as C/EBPβ, Ap2, SREBP-1c, FAS, HMGCCR, and β-actin, for a compressive knowledge of these peptides and their putative agonist action.

## Conclusion

Although raw samples (RF and RPC) have a more significant amount of protein, the GIS and particle size behavior show a significant difference (*p* < 0.05) concerning cooked samples (CF and CPC). CF and CPC show desirable physicochemical characteristics for a functional ingredient, with WAC, OAC, and emulsion capacity similar to black and cranberry common bean and a % IVDP of 62 and 64 and C-PER of 1.93 and 1.81, respectively, being slightly higher than those reported for some varieties of common bean flours, reinforcing that heat treatment is crucial to develop suitable physicochemical and nutritional properties in legume materials. In addition to the nutritional role that proteins have, the *in silico* study found that the peptides TTPW and ITY, obtained from tepary bean lectin, could exert putative antiadipogenic effects via PPAR-γ based on the intermolecular interactions formed by ligand-receptors and presence of peptides with hydrophobic amino acids such as proline, which is crucial for their functionality. However, further studies should investigate *in vitro* and *in vivo* levels to elucidate the biological activity that these peptides may present, considering the different factors that mediate the digestion of proteins like lectin as polyphenols in the food matrix and heat treatment, among others.

## Data Availability Statement

The raw data supporting the conclusions of this article will be made available by the authors, without undue reservation.

## Author Contributions

CL-I and JV-R collected the samples, performed the analysis, interpreted and analyzed the data, and wrote the manuscript, with contributions from FR-L, BR, and MB-V. JV-R along with MB-V planned the work and checked the manuscript thoroughly. BG-M and ML-C helped with funding and nutritional analysis. JB-G helped with the physicochemical analysis and the statistical analysis. All authors read and checked the manuscript properly before submission.

## Conflict of Interest

The authors declare that the research was conducted in the absence of any commercial or financial relationships that could be construed as a potential conflict of interest.
